# Conditioned Medium Reconditions Hippocampal Neurons against Kainic Acid Induced Excitotoxicity: An *In Vitro* Study

**DOI:** 10.1155/2014/194967

**Published:** 2014-11-23

**Authors:** Pradeep Kumar K. Bevinahal, Chaitra Venugopal, Harish Chandra Prasad S. Yencharla, Shashank Chandanala, Raju R. Trichur, Sathyaprabha N. Talakad, Ramesh R. Bhonde, Anandh Dhanushkodi

**Affiliations:** ^1^School of Regenerative Medicine, Manipal University, GKVK Post, Bellary Road, Allalasandra, Near Hotel Royal Orchid, Yelahanka, Bangalore 560065, India; ^2^Department of Neurophysiology, National Institute of Mental Health and Neurosciences, Deemed University, Hosur Road, Bangalore 560029, India

## Abstract

Stem cell therapy is gaining attention as a promising treatment option for neurodegenerative diseases. The functional efficacy of grafted cells is a matter of debate and the recent consensus is that the cellular and functional recoveries might be due to “by-stander” effects of grafted cells. In the present study, we investigated the neuroprotective effect of conditioned medium (CM) derived from human embryonic kidney (HEK) cells in a kainic acid (KA) induced hippocampal degeneration model system in *in vitro* condition. Hippocampal cell line was exposed to KA (200 *µ*M) for 24 hrs (lesion group) whereas, in the treatment group, hippocampal cell line was exposed to KA in combination with HEK-CM (KA + HEK-CM). We observed that KA exposure to cells resulted in significant neuronal loss. Interestingly, HEK-CM cotreatment completely attenuated the excitotoxic effects of KA. In HEK-CM cotreatment group, the cell viability was ~85–95% as opposed to 47% in KA alone group. Further investigation demonstrated that treatment with HEK-CM stimulated the endogenous cell survival factors like brain derived neurotrophic factors (BDNF) and antiapoptotic factor Bcl-2, revealing the possible mechanism of neuroprotection. Our results suggest that HEK-CM protects hippocampal neurons against excitotoxicity by stimulating the host's endogenous cell survival mechanisms.

## 1. Introduction

Glutamate mediated excitotoxicity is one of the common mechanisms of neuronal death in neurodegenerative diseases like Alzheimer's disease [1, 2]. Currently, there are no appreciable treatments available for neurodegenerative diseases and the drugs in practice provide only symptomatic relief and do not mitigate the progression of the disease. Thus, there is a pressing need to develop alternative therapeutic strategies that can prevent the progressive neuronal loss observed in neurodegenerative conditions. In this perspective, several studies have shown the beneficial effects of cell therapies in preventing neuronal loss and in reversing behavioral impairments in animal models of neurodegeneration [3–6]. However, the functional efficacy of grafted cells is still a matter of debate and the possibilities of teratoma formation and immune rejections are the major roadblocks in cell therapy. Nevertheless, the general opinion is that the host tissue regeneration may be a “by-stander” effect conferred by the grafted cells which release various trophic factors that, in turn, activate the host's endogenous reparative mechanisms [7, 8]. In this context, conditioned medium (CM) obtained from cell culture contains an array of neurotrophic factors and cytokines which might have the potential to treat neurological disorders and neurodegenerative conditions [9–11].

Human embryonic kidney (HEK) cell line is widely used for viral transfection studies and for production of therapeutic proteins by pharmaceutical companies. The benefits of HEK cells include high* in vitro* expansion and easy maintenance and are a heterogeneous population of cells including neurons [12]. Importantly, HEK conditioned medium (HEK-CM) is enriched with several growth factors [13, 14]. However, till date, the neuroprotective ability of HEK-CM* per se* against excitotoxicity has not been evaluated. In the present study, we investigated the neuroprotective effects of HEK-CM against kainic acid (KA) induced neurodegeneration in an* in vitro* model system. Our results revealed that HEK-CM confers significant neuroprotection against KA induced excitotoxicity by stimulating the host's reparative mechanisms through upregulation of cell survival factors like BDNF and Bcl-2.

## 2. Materials and Methods

### 2.1. H3 and HEK-Cell Culture

The H3 hippocampal cell line generated from postnatal day 1 hippocampus of H-2K^b^-ts A58 transgenic mice [15] was used in this study. This cell line was a generous gift from Dr. Mitradas Panicker, National Centre for Biological Sciences, Bangalore, India. The H3 cell line has been characterized [16] and shown to express neuronal markers like neurofilament-L, microtubule associated protein-2, and nestin. The H3 cells were cultured in high glucose DMEM medium (Invitrogen, Carlsbad, California, USA) containing 10% fetal bovine serum (FBS) (Hyclone, Thermo scientific, Waltham, Massachusetts, USA), 1% glutamine (Invitrogen, Carlsbad, California, USA), and 1% penicillin-streptomycin (Invitrogen, Carlsbad, California, USA) and maintained with 5% CO_2_. Similarly, HEK 293-T cells (a generous gift from Dr. Anujith Kumar, School of Regenerative Medicine, Manipal University, India) were used for obtaining conditioned medium. The HEK 293-T cells were grown in high glucose DMEM medium with 10% FBS, 1% glutamine, 1% nonessential amino acids (Invitrogen, Carlsbad, California, USA), and 1% penicillin-streptomycin and maintained with 5% CO_2_ (Hera Cell 240 CO_2_ Incubator, Thermo scientific, Carlsbad, California, USA). Upon attaining 70–80% confluency, the HEK 293-T culture medium was collected, designated as conditioned medium (CM), aliquoted, and stored at −80°C until further use (Hera Freeze, Thermo scientific, Carlsbad, California, USA).

### 2.2. Neurodegeneration and HEK-CM Treatment

Kainic acid (KA; Tocris, Bristol, UK) was used as an excitotoxin in the present study. Kainic acid was dissolved in 0.1 M phosphate buffered solution (PBS) at a concentration of 1 mg/mL. For induction of neurodegeneration, H3 culture was exposed to KA with dosages ranging from 50 *μ*M to 200 *μ*M for 24 hours. Based on the dose standardization study, we observed that 200 *μ*M of KA caused ~60% cell loss and therefore we chose this dosage for the present study. The H3 cells were plated in 96 well plates (2000 cells/well) and, on attaining 80% confluency, the cells were exposed to fresh culture medium containing 200 *μ*M KA (KA lesion group) for 24 hours. In case of conditioned medium treatment group (KA + CM group), the culture medium was replaced with HEK-CM containing 200 *μ*M KA and incubated for 24 hours. As a sham control, H3 cell culture medium was replaced with HEK-CM (CM group) and an equal volume of sterile PBS was added instead of KA. Normal control group (NC) did not receive any treatment.

### 2.3. Morphological Assessment

To study the effects of HEK-CM treatment on the morphology of H3 cells, digitized images of cultured H3 cells from different treatment groups were captured at three nonoverlapping fields using phase contrast microscopy (Nikon Eclipse TE 200-S, Chiyoda-Ku, Japan) and qualitatively examined the cell morphology for structural alterations in cell bodies, neurites, and cellular interconnections.

### 2.4. Cell Density Quantification

The H3 cells from different treatment groups were stained with nuclear staining fluorescent dye 4′,6-diamidino-2-phenylindole dihydrochloride (DAPI) diluted in sterile 0.1 M PBS and incubated for 5 minutes at room temperature. Following incubation, the cells were washed three times in PBS and digitized images were captured using fluorescence microscopy (Nikon Eclipse TE 200-S, Chiyoda-Ku, Japan) coupled with Q-Imaging camera. Q-Capture Pro 6.0 software was used to acquire images and the number of DAPI^+^ cells was quantified using Image J software (Version 1.46, NIH, USA).

### 2.5. Flow Cytometric Analysis for Apoptosis

The antiapoptotic effect of HEK-CM was assessed by flow cytometric. Briefly, H3 cells (2 × 10^6^ cells) were washed with PBS to remove debris and trypsinised with 0.25% trypsin-EDTA solution (Sigma Aldrich, St. Louis, Missouri, USA). Trypsinised cells were centrifuged at 1800 rpm (Heraeus Microfuge, Thermo scientific, Carlsbad, California, USA) for 5 minutes and the supernatant was aspirated and 900 *μ*L of 95% ice cold ethanol was added to the pellets in dropwise manner to avoid cell clumping. The cells were fixed with 95% ethanol at 4°C for 24 hours followed by several washes in PBS and 1 mL of staining solution (50 *μ*L of propidium iodide stock solution (1 mg/mL) and 50 *μ*L of RNase A stock solution (1 mL/mg)) was added to each sample and incubated in dark at 37°C for 30 minutes. Following incubation, flow-cytometry (BD FACS caliber, BD Biosciences, California, USA) was carried out and the data was analyzed using BD Cell Quest Pro, Version 5.2; BD Biosciences, California, USA.

### 2.6. Immunocytochemistry

To understand the molecular mechanisms of neuroprotection, cells from different treatment groups were fixed with 4% paraformaldehyde for 15 minutes at 4°C. Cells were washed with 0.1 M PBS twice and were permeabilized with 0.1% triton-X-100 for 15 minutes at room temperature. Following several washes in PBS, cells were incubated in blocking buffer (3% bovine serum albumin in PBS) for 30 minutes at room temperature and were incubated with primary monoclonal antibodies against brain derived neurotrophic factors (BDNF) (1 : 200; Developmental studies hybridoma bank, Iowa, USA) overnight at 4°C. The following day, cells were washed several times in 0.1 M PBS and rabbit anti-mouse secondary antibody conjugated with Alexa Fluor 568 (Life Technologies, USA) diluted in antibody diluents was added in the ratio 1 : 200 and incubated at room temperature for 1-2 hours. The cells were washed with 0.1 M PBS twice for 5 min each and a small drop of antifade mounting medium was added, cover slipped, and used for microscopic analysis. The BDNF immunostaining was carrying out in parallel with the negative control, that is, without the primary antibody. Microscopic images were captured and the quantification of corrected total cell fluorescence (CTCF) [17] was carried out using Image J software (Version 1.46, NIH, USA) and represented as fold changes against normal control.

In order to further elucidate the possible mechanisms by which HEK-CM protects neurons against excitotoxicity, neutralizing antibody against BDNF was added to the HEK-CM (HEK + CM with anti-BDNF group) and the cell viability was assessed by MTT method. Towards this, H3 cells were cultured in 96 wells plated and grown to a confluency of approximately 80%. Subsequent to that, the cells were treated with HEK-CM (CM group), kainic acid (KA group), Kainic acid and HEK-CM (KA + CM group), and Kainic acid and HEK-CM with anti-BDNF (HEK + CM with anti-BDNF group) for 24 hours in 5% CO_2_ incubator. Following that, cells were washed in PBS and incubated with MTT reagent (5 mg/mL, Sigma Aldrich, St. Louis, Missouri, USA) for 4 hours until a purple precipitate was formed. Next, the precipitates were dissolved with 10 *μ*L of dimethyl sulfoxide (Sigma Aldrich, St. Louis, Missouri, USA) and the optical density was measured at 560 nm using Victor^3^ multilabel plate reader (Perkin Elmer, USA). As a blank control, three wells with only culture medium plus MTT were used.

### 2.7. Reverse Transcriptase Polymerase Chain Reaction (RT-PCR)

As BDNF is known to induce the expression of antiapoptotic factors like Bcl-2, we estimated the mRNA expression of Bcl-2 in H3 cells following different treatments. To estimate the mRNA expression of Bcl-2, RNA was extracted (TRIzol, Invitrogen Life Technologies, USA) and the purity/concentration of RNA was estimated using Nano Drop ND-1000 spectrophotometer (Nanodrop Technologies Inc. Wilmington, Delaware, USA). Using RNA, cDNA was synthesized and PCR amplification (GeneAmp PCR system 9700, Applied Biosystems Inc, Foster City, California, USA) was carried out in accordance with the standard protocol for 40 cycles using 55–60°C as annealing temperature. G3PHD was used as an internal control. The following primers were used in this study: G3PDH (forward: 5′TCCACCACCCTGTTGCTGTA3′, reverse: 5′ACCACAGTCCATGCCATCAC3′), Bcl-2 (forward: 5′CACCTGTGGTCCACCTGAC3′, reverse: 5′ACGCTCTCCACACACATGAC3′). Amplified PCR products were gel electrophoresed and visualized by Alpha Imager gel documentation system (Alpha Imager Gel Documentation, Alpha Innotech, San Leandro, California, USA).

### 2.8. Statistical Analysis

Three independent experiments were carried out in triplicate for cell density analysis, immunocytochemistry, and MTT assay. Two independent experiments were carried out for flow cytometer and RT-PCR analysis. All data are presented as means ± SEM. For statistical comparisons between different treatment groups, one way analysis of variance followed by Newman-Keuls multiple comparison test was used and *P* < 0.05 was considered as significant.

## 3. Results

### 3.1. HEK-CM Treatment Improves the Morphology of H3 Cells

**Figure 1:**
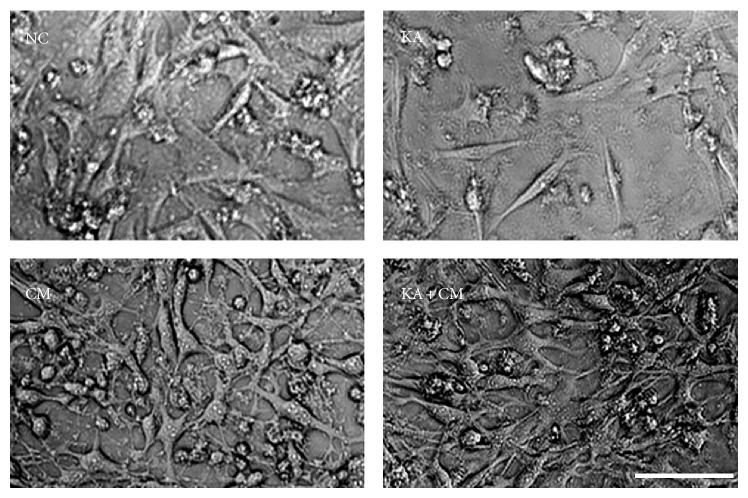
Neuromorphological alteration. Representative photomicrographes demonstrating the morphology of H3 cells in different treatment groups. Kainic acid treatment to H3 cells resulted in prominent decrease in cell density and neurites. In contrast, KA + CM group demonstrated healthy cell bodies with dense neurites. Scale Bar 100 *μ*m. Normal control (NC), normal control exposed to HEK conditioned medium (CM), Kainic acid alone treated (KA), and Kainic acid and HEK conditioned medium cotreated (KA + CM).

To investigate the neuroprotective effect of HEK-CM against KA induced excitotoxicity, H3 cells were exposed to KA alone (200 *μ*M) or in combination with HEK-CM (KA + CM) for 24 hours. Following that, cell culture images were acquired at three nonoverlapping fields and the morphological features of H3 cells were analyzed quantitatively. Kainic acid exposure to H3 cells resulted in prominent reduction in neurites and cell density (Figure 1). On the other hand, exposure of HEK-CM to normal control and KA treated cells displayed healthy cell bodies and enhanced neuronal processes.

### 3.2. HEK-CM Treatment Preserves Cell Density

**Figure 2:**
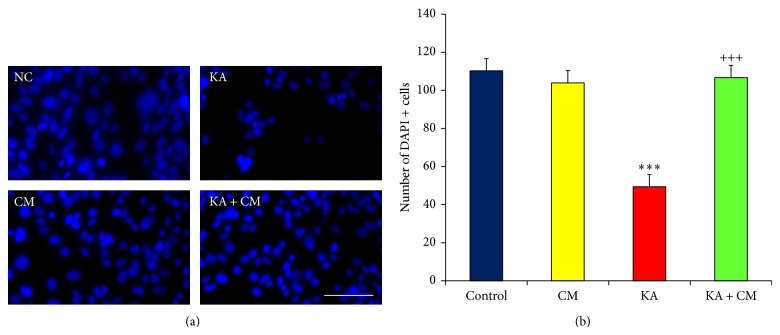
HEK-CM protects neurons. (a) Photomicrograph of DAPI^+^ cells representing cell density in different treatment groups. (b) Bar diagrams representing the number of viable cells as quantified by DAPI^+^ cells. Scale Bar 200 *μ*m. Normal control (NC), normal control exposed to HEK conditioned medium (CM), Kainic acid alone treated (KA), and Kainic acid and HEK conditioned medium cotreated (KA + CM). Data represented as mean ± SEM. ^*^ indicates comparison between NC and treatment; ^+^ indicates comparison between KA lesion and treatment; ^***^ *P* < 0.001; ^+++^ *P* < 0.001.

To quantify the number of viable cells in different treatment groups, H3 cells were stained with nuclear dye DAPI and microscopic images were captured and quantified using Image J software. Quantification of DAPI^+^ cells from different treatment groups revealed that KA exposure resulted in significant cell loss (*P* < 0.001; Figures 2(a) and 2(b)) as the cell density was 49.4 ± 7.2 as compared to normal control group (110 ± 6.42). Conversely, exposure of KA with HEK-CM completely protected the neurons from KA induced excitotoxicity and the cell density was significantly higher in KA + CM group (106.7 ± 4.65; *P* < 0.001, Figures 2(a) and 2(b)) as compared to KA alone group (49.4 ± 7.2). HEK-CM medium* per se* did not reduce the cell density in the CM group as the cell density (103.9 ± 3.14; Figures 2(a) and 2(b)) was comparable to that of the normal control group.

### 3.3. Antiapoptotic Potential of HEK-CM

**Figure 3:**
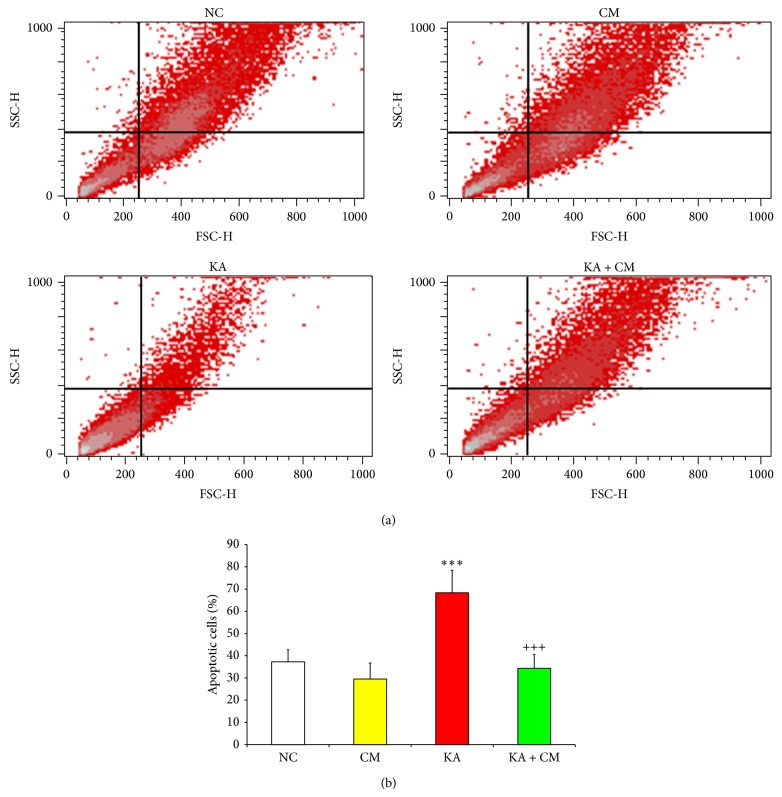
Antiapoptotic effect of HEK-CM. (a) Representative Flow cytometric Scatter Plots demonstrating the apoptotic and antiapoptotic effects of KA and HEK-CM, respectively. (b) Bar diagrams representing the percentage of apoptotic cells in various treatment groups. Normal control (NC), normal control exposed to HEK conditioned medium (CM), Kainic acid alone treated (KA), and Kainic acid and HEK conditioned medium cotreated (KA + CM). Data represented as mean ± SEM. ^*^ indicates comparison between NC and treatment; ^+^ indicates comparison between KA lesion and treatment; ^***^ *P* < 0.001; ^+++^ *P* < 0.001.

Next, we ascertain the percentage of apoptotic cells in different treatment groups by flow cytometric analysis. In KA 24 hrs group, there was a significant increase in percentage of apoptotic cells (83%) as compared to NC group (*P* < 0.001; Figure 3). On the other hand, when cells were cotreated with KA and HEK-CM, a significant antiapoptotic effect was observed and there was a 50% percentage decrease in apoptotic cells as compared to KA 24 hrs group (*P* < 0.001; Figure 3) suggesting that HEK-CM protects neurons against excitotoxicity by antiapoptotic mechanism.

### 3.4. HEK-CM Enhances Host's BDNF Level

**Figure 4:**
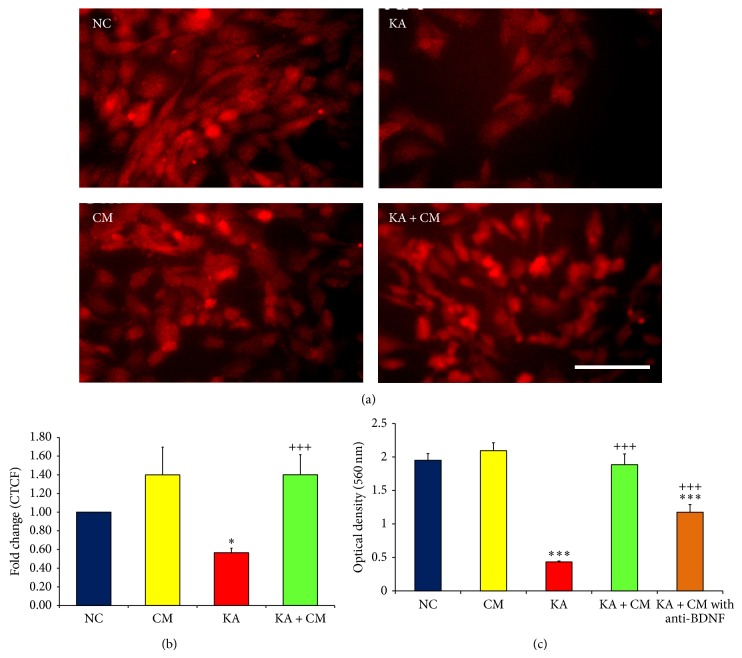
HEK-CM stimulates host's cell survival factors BDNF and Bcl-2. (a) Photomicrograph represents the expression of BDNF in different treatment groups. Scale Bar 200 *μ*m. (b) Bar diagram signifies the fold changes in BDNF fluorescence intensity as compared to NC. (c) MTT Assay. Bar diagram represents the viable cells in different treatment groups. Neutralization of BDNF in HEK-CM diminishes the neuroprotective potential of HEK-CM against excitotoxicity. Nevertheless, significant neuroprotection was observed in KA + CM with anti-BDNF group as compared to KA group suggesting that other growth factors/cytokines in CM might be contributing to the observed neuroprotection. Normal control (NC), normal control exposed to HEK conditioned medium (CM), Kainic acid alone treated (KA), Kainic acid and HEK conditioned medium cotreated (KA + CM), and Kainic acid and BDNF neutralized HEK conditioned medium cotreated (KA + CM with anti-BDNF). Data represented as mean ± SEM. ^*^ indicates comparison between NC and treatment; ^+^ indicates comparison between KA lesion and treatment; ^*^ *P* < 0.05; ^***^ *P* < 0.001; ^+++^ *P* < 0.001.

Brain derived neurotrophic factor (BDNF) plays an important role in neuronal survival, proliferation, and neurites growth. In order to gain mechanistic insight into the neuroprotective potential of HEK-CM, we analyzed the expression of BDNF in host cells (H3 cells) from different treatment groups. Cells from various treatment groups were processed for BDNF immunocytochemistry and the fluorescence intensity was measured using Image-J software and expressed as corrected total cell fluorescent (CTCF) [17]. Following KA exposure, there was a 1.7-fold decrease in BDNF expression in surviving H3 cells as compared to normal control group (*P* < 0.05; Figures 4(a) and 4(b)). HEK-CM enhanced the H3 cell's BDNF expression in both CM and KA + CM group. The BDNF expression was 2.4-fold higher in the KA + CM group as compared to KA alone group (*P* < 0.001; Figures 4(a) and 4(b)). Likewise, there was 1.4-fold increase in BDNF expression in the CM group compared to normal control group. However, it was not statistically significant (Figures 4(a) and 4(b)).

Furthermore, the contribution of BDNF in HEK-CM for neuroprotection was determined by adding BDNF neutralizing antibody to the HEK-CM and the cell viability was assessed by MTT assay. Our data revived that a significant portion of neuroprotective potential of HEK-CM was diminished when the BDNF in HEK-CM was neutralized (Figure 4(c)). Neutralization of BDNF in HEK-CM significantly reduced the neuroprotective potential of HEK-CM from 96% to 60% (*P* < 0.001; Figure 4(c)). Interestingly, in spite of inactivating the BDNF in HEK-CM with neutralizing antibody, we still observed ~40% of neuroprotection (*P* < 0.001; Figure 4(c)) in HEK + CM with anti-BDNF group as compared to KA alone group suggesting that there may be other growth factors in HEK-CM that might also be contributing to the neuroprotective effects observed.

### 3.5. HEK Treatment Increases the mRNA Levels of Bcl-2

**Figure 5:**
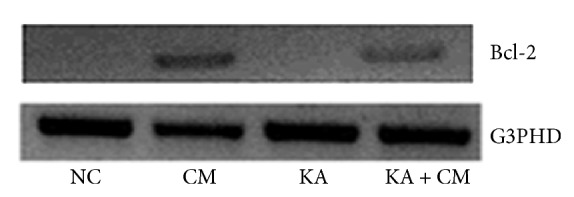
Messenger RNA expression of antiapoptotic factor Bcl-2 in different treatment groups. Normal control (NC), normal control exposed to HEK conditioned medium (CM), Kainic acid alone treated (KA), and Kainic acid and HEK conditioned medium cotreated (KA + CM).

As BDNF exerts its neuroprotective effects through upregulation of antiapoptotic factors like Bcl-2, we investigated the expression of endogenous antiapoptotic factor like Bcl-2 at mRNA level by RT-PCR method. Our results demonstrated that exposure of HEK-CM to normal control as well as KA + CM group resulted in prominent upregulation of Bcl-2 expression at the mRNA level (Figure 5). In contrast, we could not detect any Bcl-2 expression in KA treated and normal control groups (Figure 5).

## 4. Discussion

In the present study, we demonstrate that conditioned medium derived from HEK cells can protect hippocampal neurons against kainic acid induced excitotoxicity. We further show that the neuroprotection mediated by CM is attributed to the stimulation of endogenous cell survival factors like BDNF and antiapoptotic factor Bcl-2 in the host cells. The underlying cause of neuronal loss in neurodegenerative diseases is mainly due to high extracellular concentration of glutamate released by degenerating neurons and the prolonged activation of glutamate receptors on neurons surrounding the lesion vicinity [18]. Hyperactivation of glutamate receptors leads to disturbances in calcium homeostasis, altered mitochondrial potential, and activation of downstream cell death pathways [1, 19, 20]. In neurodegenerative conditions, two known cell death pathways, namely, necrosis and apoptosis, are activated wherein the necrotic cell death occurs during the initial injury and apoptotic cell death contributes to the progressive neuronal loss which is typically observed in many neurodegenerative diseases. In the present study, we used an* in vitro* model of hippocampal neurodegeneration by exposing toxic dose of glutamate analogue kainic acid [21] to H3 hippocampal cells. Kainic acid exposure at a dosage of 200 *μ*M for a duration of 24 hours significantly reduced the cell viability and concurrently increased apoptosis in remaining H3 cells. Conversely, the neural loss mediated by KA was completely rescued when H3 cells were cotreated with HEK-CM. Previous studies have demonstrated that coculturing neuronal cells with bone marrow derived mesenchymal stem cells (BM-MSC) can protect neurons against excitotoxicity by altering the expression of glutamate receptors [22] and enhance neuronal survival by cell-cell contact [23]. Interestingly, a recent report by Voulgari-Kokota et al., [22] demonstrated that pretreatment with conditioned medium derived from bone marrow MSCs (BM-MSC-CM) could prevent retinal ganglion neurons from glutamate mediated excitotoxicity. Similarly, pretreatment of BM-MSC-CM has also been reported to protect rat embryonic neurons against staurosporine/amyloid beta peptide induced neuronal death [24]. In the later study, the BM-MSC-CM mediated neuroprotection was observed when BM-MSC-CM was used at a dose of 30–50%, whereas 100% of BM-MSC-CM exposure resulted in elevated neuronal apoptosis. Furthermore, neuroprotection was observed only in pretreated group whereas BM-MSC-CM cotreatment did not protect the neurons from staurosporine/amyloid beta peptide induced cell death. Interestingly, posttreatment of conditioned medium derived from adipose MSCs could also protect PC-12 neural cells against glutamate mediated excitotoxicity [25]. In the present study, we observed that cotreatment of HEK-CM offered significant neuroprotection against KA induced excitotoxicity suggesting that the neuroprotective ability of conditioned medium depends on the source of conditioned medium used and the neurotoxin applied to induce neurodegeneration.

In the field of cytotherapy, several hypotheses were put forth to explain the possible mechanisms of cellular and functional recoveries following cell transplantation that includes differentiation of grafted cells into local phenotypes [26, 27], graft cell-host cell fusion [28], and secretion of growth factors and cytokines that can induce host's endogenous reparative mechanisms [8]. Among these, secretome mediated tissue regeneration has attracted more attention in recent years [5, 9–11]. Furthermore, several studies have shown significant functional recovery in spite of poor engraftment of transplanted cells, thus supporting the notion that the grafted cells either release or stimulate the expression of various endogenous cell survival factors for functional recoveries [29]. In line with this, we have also observed that HEK-CM* per se* can induce the expression of host cell survival factors like BDNF significantly. Following HEK-CM treatment, there were 2.4- and 1.4-fold increase in BDNF expression in KA treated and non-KA treated group, respectively, suggesting that CM can stimulate the expression of endogenous cell survival factors in normal and degenerative condition. Brain derived neurotrophic factor confers neuroprotection through activation of cell survival signaling that ultimately results in upregulation of antiapoptotic factors like Bcl-2. In our present study, we observed that CM treatment resulted in enhanced expression of host's Bcl-2 mRNA in KA treated as well as non-KA treated groups. In support of our findings, a recent study using CM derived from adipose-MSCs reported that CM* per se* can exert significant neuroprotection against glutamate toxicity by enhancing the level of neurotrophic factors and activating the cell survival pathway (PI3-K/Akt) that eventually results in upregulation of antiapoptotic factors like Bcl-2 and x-chromosome linked inhibitor of apoptosis [25]. Furthermore, it is unlikely that the presence of a single growth factor like BDNF could confer complete neuroprotection. As HEK-CM possesses several other growth factors that might have acted in a synergistic manner in protecting neurons against kainic acid induced cell death. Further studies are warranted to identify various growth factors in HEK-CM and to dissect out the role of individual growth factor's contribution for neuroprotection.

## 5. Conclusion

In the present study, using an* in vitro* model of hippocampal neurodegeneration, we report that conditioned medium* per se* can protect neurons against excitotoxicity by enhancing the host's endogenous cell survival mechanisms, thus highlighting the possibilities of cell therapy without cells.
